# β-Galactosidase is a target enzyme for detecting peritoneal metastasis of gastric cancer

**DOI:** 10.1038/s41598-021-88982-2

**Published:** 2021-05-21

**Authors:** Hidemasa Kubo, Yasutoshi Murayama, Soichiro Ogawa, Tatsuya Matsumoto, Masayuki Yubakami, Takuma Ohashi, Takeshi Kubota, Kazuma Okamoto, Mako Kamiya, Yasuteru Urano, Eigo Otsuji

**Affiliations:** 1 Division of Digestive Surgery, Department of Surgery, Kyoto Prefectural University of Medicine, 465 Kajii-cho, Kamigyo-ku, Kyoto 602-8566 Japan; 2 Graduate School of Medicine, The University of Tokyo, 7-3-1 Hongo, Bunkyo-ku, Tokyo 113-0033 Japan; 3 Graduate School of Pharmaceutical Sciences, The University of Tokyo, 7-3-1 Hongo, Bunkyo-ku, Tokyo 113-0033 Japan; 4 CREST (Japan) Agency for Medical Research and Development (AMED), 1-7-1 Otemachi, Chiyoda-ku, Tokyo 100-0004 Japan

**Keywords:** Gastric cancer, Metastasis, Surgical oncology, Translational research

## Abstract

Diagnosis of peritoneal metastasis in gastric cancer (GC) is essential for determining appropriate therapeutic strategies and avoiding non-essential laparotomy or gastrectomy. Recently, a variety of activatable fluorescence probes that can detect enzyme activities have been developed for cancer imaging. The aim of this study was to identify the key enzyme involved in peritoneal metastasis in GC. The enzymatic activity of gamma-glutamyl transpeptidase, dipeptidyl peptidase IV, and β-galactosidase (β-Gal) was assessed in lysates prepared from preserved human GC (n = 89) and normal peritoneal (NP; n = 20) samples. β-Gal activity was significantly higher in the human GC samples than in NP samples, whereas no differences were observed in the activities of the other enzymes. Therefore, we used SPiDER-βGal, a fluorescent probe that can be activated by β-Gal, for imaging GC cell lines, peritoneal metastasis in a mouse model, and fresh human resected GC samples (n = 13). All cell lines showed fluorescence after applying SPiDER-βGal, and metastatic nodules in the mice gradually developed high fluorescence that could be visualized with SPiDER-βGal. The human GC samples showed significantly higher fluorescence than NP samples. β-Gal is a useful target enzyme for fluorescence imaging of peritoneal metastasis in GC.

## Introduction

Gastric cancer (GC) is one of the most common types of cancer worldwide^1^, and peritoneal metastasis is an important prognostic factor. Peritoneal metastasis is considered to be non-curable^2^, and gastrectomy for advanced GC patients with peritoneal metastasis did not show a survival benefit in phase III randomized controlled trials^3^. Thus, an accurate diagnosis of peritoneal metastasis is essential for determining the appropriate therapeutic strategies and avoiding laparotomy or gastrectomy whenever possible.

Staging laparoscopy is more useful for the diagnosis of peritoneal metastasis compared to computed tomography (CT) with a sensitivity of 64–94% and 33%, respectively^4–6^. However, the probability of obtaining false-negative results with staging laparoscopy is approximately 10.6–17.2%^7–9^. Although false-negative cases are clinically important, cases with tiny nodules, nodules inside of the bursa, or nodules where the shape of the metastasis is not formed may be missed completely.

In recent years, various fluorescence techniques have been developed to improve the diagnostic results for peritoneal metastasis. For example, photodynamic diagnosis using 5-aminolevulinic acid (5-ALA) and indocyanine green (ICG) is useful in detecting peritoneal metastasis^10–12^. However, both 5-ALA and ICG need to be administered prior to surgery. Moreover, a variety of activatable fluorescence probes targeting cancer-associated enzymes have been developed^13–15^. These probes are originally nonfluorescent; however, after an enzymatic reaction, they become highly fluorescent within a few minutes. Thus, malignancy can be rapidly detected in cancer samples simply by topically spraying the activatable probes targeting the cancer-associated enzymes. For example, gGlu-HMRG, a gamma-glutamyl transpeptidase (GGT)-activatable fluorescent probe, can detect breast cancer^16^, lung cancer^17^, hepatocellular carcinoma^18^, and lymph node metastasis of colorectal cancer^19^. Furthermore, a dipeptidyl peptidase IV (DPPIV)-activatable fluorescence probe can detect esophageal cancer^14^. Moreover, SPiDER-βGal, which is activated by β-galactosidase (β-Gal), has been tested on ovarian cancer cell lines^20,21^. However, it is important to select specific target enzymes for each cancer type according to its upregulated expression in the cancer tissue compared with that in the surrounding normal tissue to achieve specific fluorescence imaging.

Since there is no known target enzyme, fluorescence imaging of peritoneal metastasis in GC by activatable fluorescence probes has not been reported to date. Thus, the aim of the present study was to find a suitable target enzyme and a compatible enzyme-activatable fluorescence probe to detect peritoneal metastasis in GC.

## Results

### In vitro β-Gal activity of human GC samples

**Figure 1:**
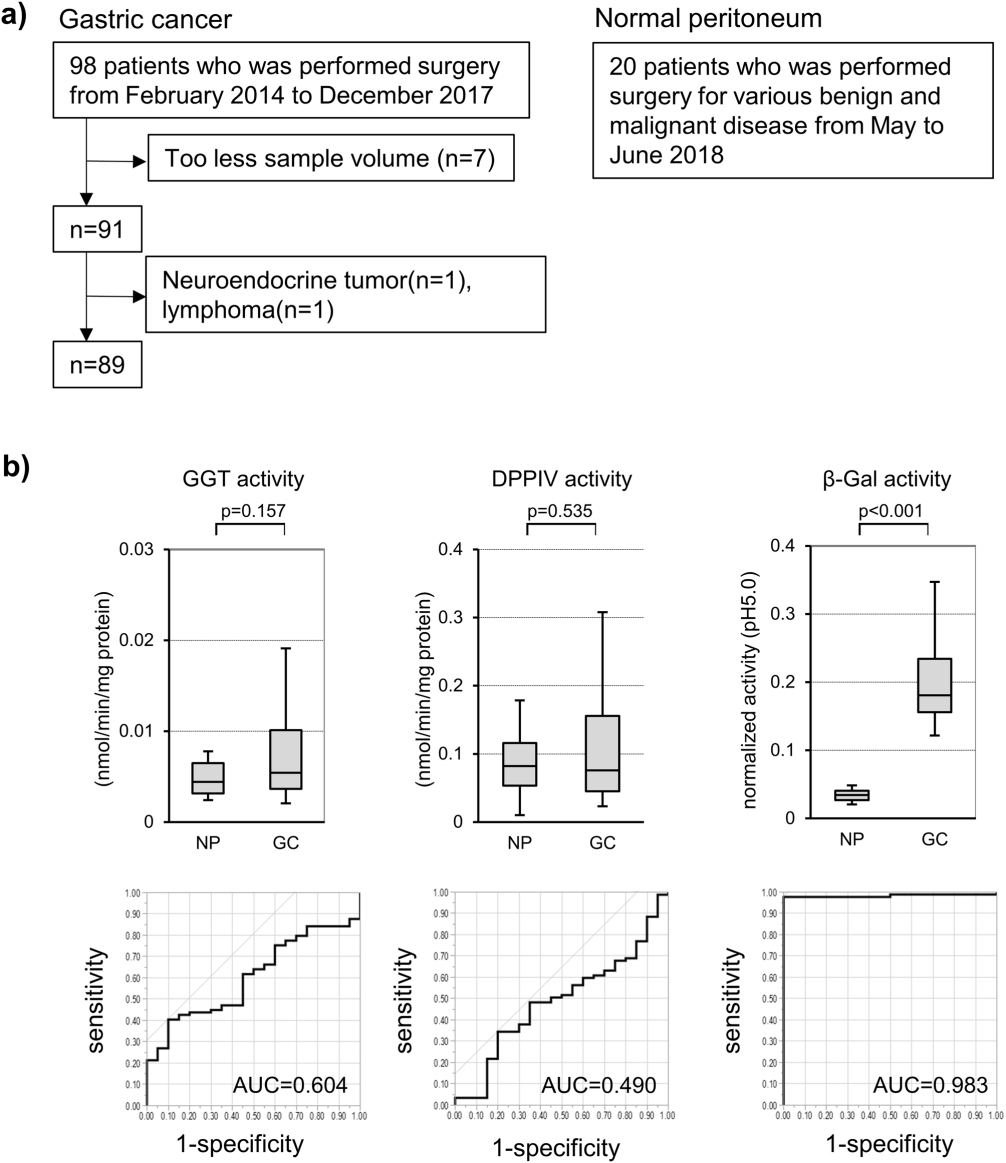
GGT, DPPIV, and β-Gal activity of preserved tumor and NP samples. (**a**) Consort diagram of GC and NP samples used in this study. (**b**) GGT, DPPIV, and β-Gal activity and ROC of NP (n = 20) and GC (n = 89). GGT, gamma-glutamyl transpeptidase; DPPIV, dipeptidyl peptidase IV; β-Gal, β-galactosidase; GC, gastric cancer; NP, normal peritoneum; ROC, receiver operating characteristic curve; AUC, area under the curve.

We prepared lysates of preserved human GC (n = 89) and NP (n = 20) samples (Fig. 1a). The enzymatic activities of GGT, DPPIV, and β-Gal in the GC and NP lysates were assessed. There were no significant differences between the activity of GGT and DPPIV. By contrast, the normalized β-Gal activity of NP and GC was 0.035 (range: 0.012–0.061) and 0.181 (range: 0.002–0.516), respectively (*p* < 0.001) in pH 5.0 (Fig. 1b). Overall, the β-Gal activity at pH 7.4 was lower than that at pH 5.0. However, regardless of pH, a clear and similar difference in β-Gal activity was observed between GC and NP samples (Supplementary Fig. 1), such that the β-Gal activity of GC samples was significantly higher than that of NP samples. The area under the ROC curve (AUC) was 0.983 (Fig. 1b). These results showed that β-Gal could be a candidate target enzyme for the fluorescence imaging of peritoneal metastasis of GC.

There was no significant difference in the β-Gal activity according to various clinicopathological characteristics of the patients (Table 1).

**Table 1:** β-Gal activity of each clinicopathological factor.

Variables (n = 89)		n	β-Gal activity	*p*-value
Sex	Male	68	0.181(0.002–0.516)	0.546
	Female	21	0.181(0.034–0.444)	
Age (years)	< 75	55	0.175(0.002–0.516)	0.057
	≥ 75	34	0.191(0.111–0.463)	
Preoperative BMI (kg/m^2^)	< 25	67	0.179(0.034–0.463)	0.816
	≥ 25	22	0.183(0.002–0.516)	
Preoperative Hb (g/dl)	< 12.0	29	0.188(0.111–0.463)	0.129
	≥ 12.0	60	0.175(0.002–0.516)	
Preoperative albumin (g/dl)	< 4.0	36	0.186(0.104–0.463)	0.237
	≥ 4.0	53	0.176(0.002–0.516)	
Preoperative CRP (mg/dl)	< 0.3	71	0.181(0.002–0.463)	0.818
	≥ 0.3	18	0.175(0.104–0.516)	
Preoperative CEA (ng/dl)	< 5.0	53	0.179(0.002–0.463)	0.637
	≥ 5.0	36	0.184(0.093–0.516)	
Preoperative CA19-9 (U/ml)	< 37	75	0.182(0.002–0.516)	0.723
	≥ 37	14	0.173(0.121–0.463)	
NAC	−	82	0.182(0.002–0.516)	0.154
	+	7	0.161(0.104–0.272)	
pT	1–2	31	0.182(0.002–0.516)	0.483
	3–4	58	0.178(0.034–0.463)	
pN	−	23	0.188(0.104–0.456)	0.407
	+	66	0.178(0.002–0.516)	
ly	−	15	0.188(0.115–0.463)	0.453
	+	74	0.180(0.002–0.516)	
v	−	17	0.182(0.115–0.456)	0.635
	+	72	0.180(0.002–0.516)	
Histological type	dif	50	0.184(0.002–0.463)	0.435
	undif	39	0.175(0.104–0.516)	

Values of *p* were obtained by Wilcoxon test. *BMI* body mass index, *CRP* C-reactive protein, *CEA* Carcinoembryonic antigen, *CA19-9* carbohydrate antigen 19-9, *NAC* neoadjuvant chemotherapy, *dif* differentiated, *undif* undifferentiated.

### Fluorescence imaging study of GC cell lines

Next, we planned a fluorescence imaging study using GC cell lines and SPiDER-βGal. SPiDER-βGal is an activatable fluorescence probe that is originally nonfluorescent. However, it emits high fluorescence after cleavage by β-Gal.

**Figure 2:**
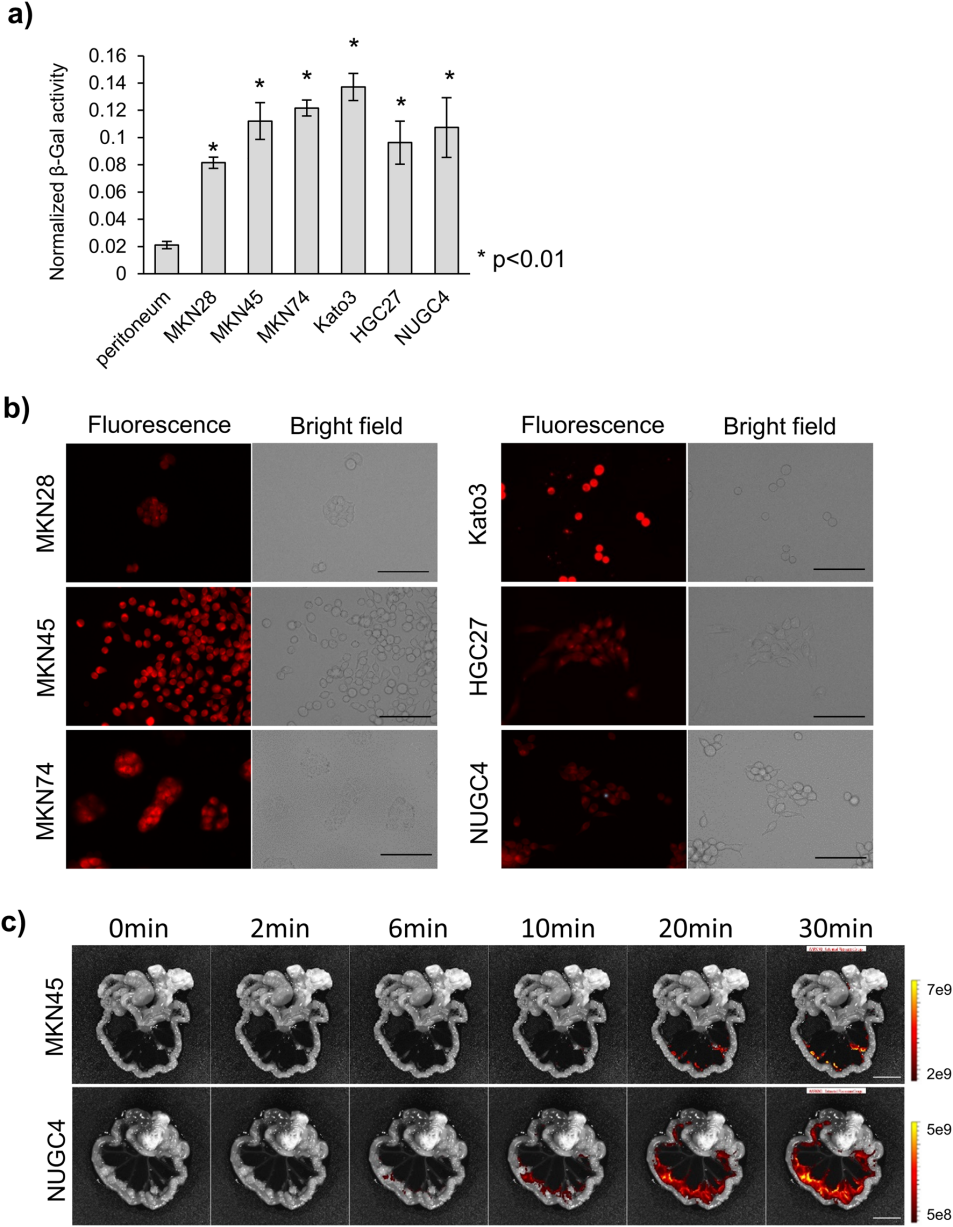
GC cell line analyses. (**a**) β-Gal activity of the GC cell line lysates and mouse NP. (**b**) Live cell imaging of GC cell lines. Scale bar, 100 μm. (**c**) Ex vivo imaging of the mouse model of peritoneal metastasis. Scale bar, 10 mm. β-Gal, β-galactosidase; GC, gastric cancer; NP, normal peritoneum.

First, we prepared lysates of six GC cell lines, MKN28, MKN45, MKN74, Kato3, HGC27, and NUGC4, and mouse NP prior to conducting live cell imaging and imaging of a peritoneal metastasis mouse model. We assessed the β-Gal activity of these GC cell lines, and NP was used as a control (Fig. 2a). The β-Gal activity of all GC cell lines was significantly higher than that of the mouse NP. Next, we performed live cell imaging of the GC cell lines by using SPiDER-βGal. All cell lines showed fluorescence after applying SPiDER-βGal (Fig. 2b). Further, we established a mouse model of peritoneal metastasis in GC by injecting the cell lines into the mouse abdominal cavity. MKN45 and NUGC4 cells formed peritoneal metastases, and ex vivo imaging was carried out by spraying SPiDER-βGal. Metastatic nodules gradually developed high fluorescence and could be visualized (Fig. 2c).

### Ex vivo imaging of freshly resected human GC samples

**Figure 3:**
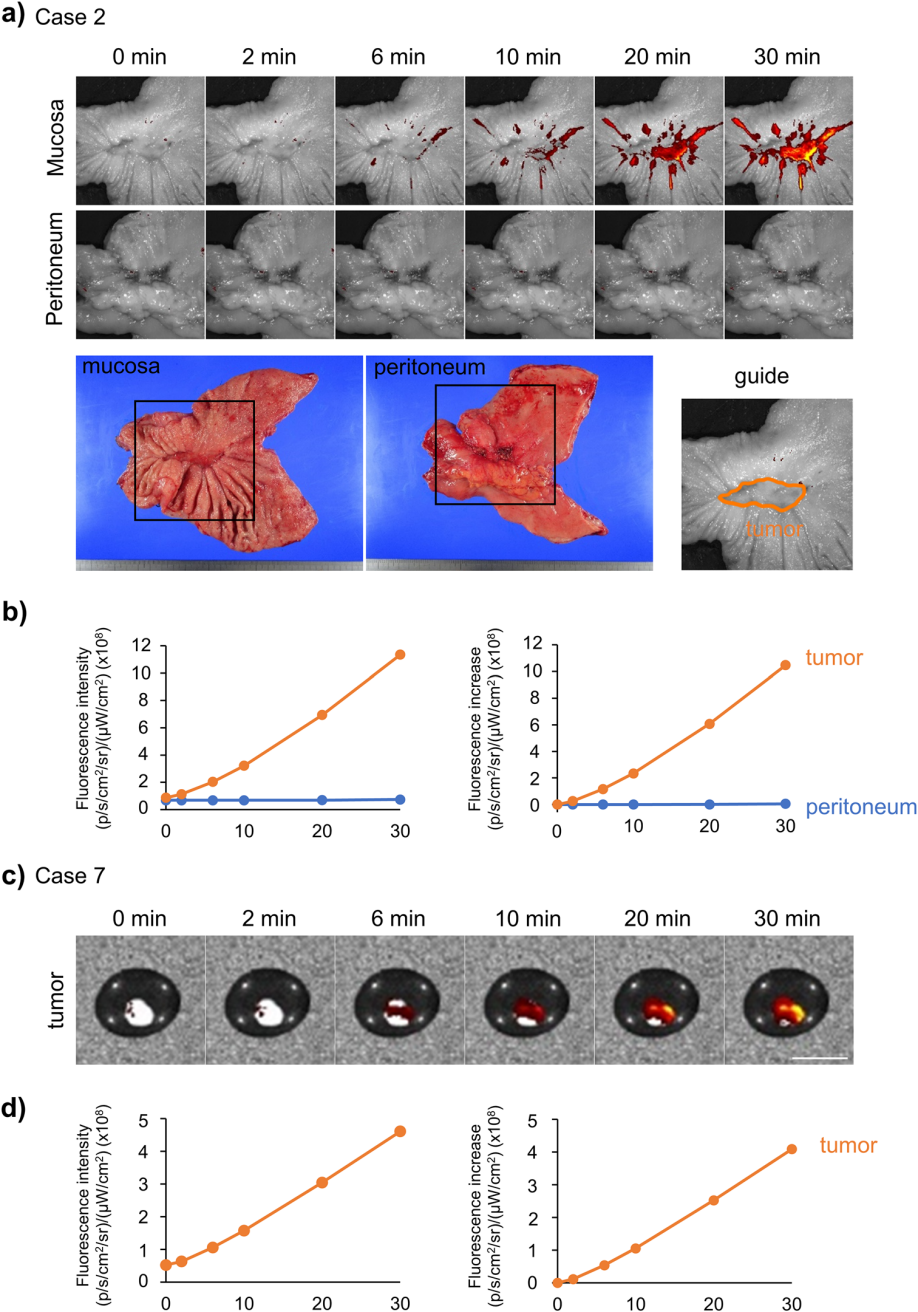
A representative case of fluorescence imaging of fresh human resected GC samples. (**a**) Fluorescence imaging of the mucosa and peritoneum sides with SPiDER-βGal. (**b**) Time course of fluorescence intensity and increase in the tumor and peritoneum. (**c**) Fluorescence imaging of peritoneal metastatic nodules with SPiDER-βGal. Scale bar, 10 mm. (**d**) Time course of fluorescence intensity and increase in the nodules.

Based on these results, we performed fluorescence imaging of freshly resected human GC samples. We sprayed SPiDER-βGal on both the mucosal and peritoneal sides of the resected human stomach; the visceral peritoneum was used as a control. Representative cases (cases 2 and 7) are shown in Fig. 3. All the other cases are shown in Supplementary Fig. 2. The fluorescence intensity of the tumor in Case 2 was higher than that of the peritoneum (Fig. 3a, b). In Case 7, we conducted fluorescence imaging of peritoneal metastatic nodules by spraying SPiDER-βGal. Although there was no control, the metastatic nodule showed increased fluorescence over time; the fluorescence intensity after 30 min was approximately eight times higher than that at 0 min (Fig. 3c, d).

**Figure 4:**
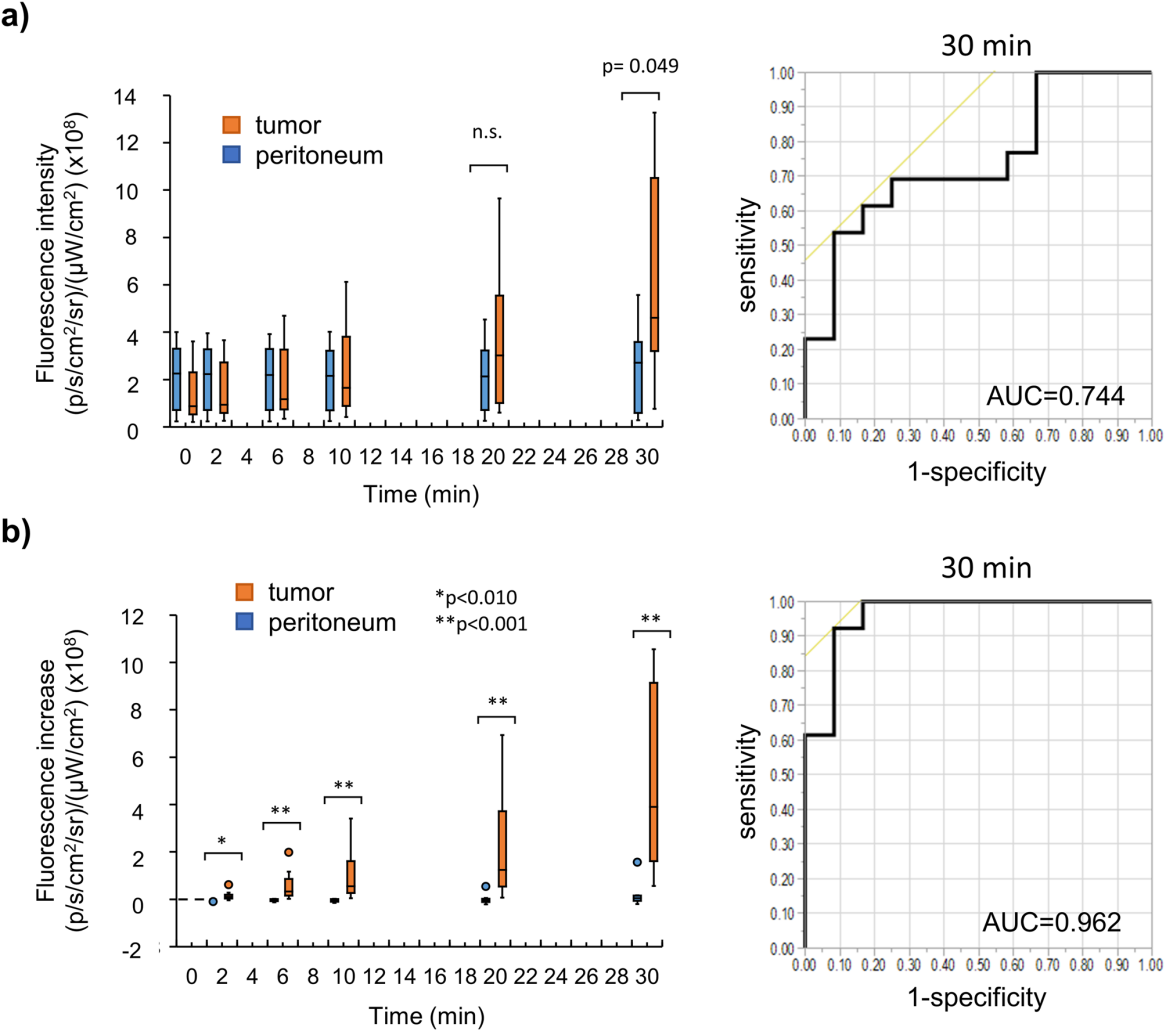
(**a**) Time course of fluorescence intensity of the tumor and peritoneum in all cases and ROC at 30 minutes after spraying SPiDER-βGal. (**b**) Time course of the fluorescence increase in the tumor and peritoneum in all cases and ROC at 30 minutes after spraying SPiDER-βGal. β-Gal, β-galactosidase; ROC, receiver operating characteristic curve; n.s., not significant.

We evaluated a total of 13 cases (including the above 2 cases). The patient characteristics of these 13 cases are listed in Table 2. In 12 cases, the mucosal and peritoneal sides of primary GC were assessed. The peritoneal metastatic nodules were assessed in only one case (Case 7). The tumor in Case 1 was a pancreatic invasion; however, it was not exposed during the imaging of the peritoneal side. In all cases, we compared the fluorescence intensities between the tumor and peritoneum (Fig. 4a). Thirty minutes after spraying, the fluorescence intensity was significantly higher in the tumor than in the peritoneum. The AUC of the ROC curve was 0.744. In all cases, there was also a slight increase in the fluorescence of the peritoneum; however, the fluorescence increase of the tumor from 0 min was much greater. The AUC of the ROC curve was 0.962 (Fig. 4b).

**Table 2:** Patient characteristics of each case in clinical sample imaging.

	Sex	Age	Borrmann type	Histology	Size (mm)	Depth	ly	v	N	NAC	Effect of NAC
Case 1	M	65	3	tub2	80 × 40	si(panc)	0	2	+	+	1a
Case 2	M	70	3	por2	34 × 22	mp	1c	0	−	+	2a
Case 3	M	68	2	tub1	44 × 34	ss	1a	1b	+	−	
Case 4	F	76	0-IIc	sig	48 × 47	se	1a	1a	+	−	
Case 5	M	73	3	por2	45 × 15	mp	1c	1c	+	−	
Case 6	F	76	4	por2	66 × 59	se	1c	1c	+	−	
Case 7	M	79	Adenocarcinoma								
Case 8	M	87	2	por1	57 × 48	ss	0	1c	+	−	
Case 9	F	81	3	por2	34 × 22	mp	1a	0	−	−	
Case 10	M	85	1	tub1	81 × 66	ss	1c	1c	+	−	
Case 11	M	49	2	muc	38 × 29	ss	1a	1b	−	−	
Case 12	F	78	0-IIa + IIb	tub1	35 × 20	sm	0	1	+	−	
Case 13	F	75	4	por2	14 × 12	mp	0	0	−	−	

*NAC* neoadjuvant chemotherapy.

## Discussion

Here, we revealed that the β-Gal activity in GC is significantly higher than that of the NP, suggesting the potential of β-Gal to serve as a target enzyme for the development of an activatable fluorescence probe for imaging peritoneal metastasis in GC. We confirmed that SPiDER-βGal is useful for sample imaging of GC using fluorescence imaging of GC cell lines, a mouse model of peritoneal metastasis, and fresh human GC samples.

Activatable fluorescence probes are useful for detecting various cancers^14,15–21^. However, there are no reports on the use of such probes for the detection of peritoneal metastasis in GC. To apply an activatable fluorescence probe for cancer imaging, a target enzyme that is specifically overexpressed in each cancer needs to be identified. In this study, we investigated potential candidate enzymes for detecting peritoneal metastasis by assaying the enzymatic activity of lysates prepared from preserved clinical samples.

β-Gal activity has been reported to be upregulated in some malignancies^22–24^; however, there are few reports in GC. In this study, the theoretical reason why β-Gal activity in GC is significantly higher than that of NP was not determined. β-Gal is known as a senescence marker^25^, and SPiDER-βGal is also used for detecting senescence-associated β-Gal (SA-β-Gal)^26^. Recently, the association between cellular senescence and cancer has been reported^27^. Hence, we suggest that SA-β-Gal might be over-expressed in GC cells which enables their detection by SPiDER-βGal.

β-Gal is a lysosomal enzyme; thus, the assessment of the β-Gal activity of the lysate was considered to be appropriate at pH 5.0^15^. However, since the resected samples did not have a blood supply, the pH of the cells and their organelles in these samples was unclear. In the lysate assay, we assessed the β-Gal activity at not only pH 5.0 but also at pH 7.4. In both acidic and neutral environments, the β-Gal activity of GC was significantly higher than that of NP. Thus, the difference between the tumor and NP was significant regardless of the pH of the samples. This was further validated in the imaging of clinical samples using SPiDER-βGal.

We imaged 12 clinical samples of the primary tumors and visceral peritoneum, demonstrating higher autofluorescence in the peritoneum than in the tumors before spraying SPiDER-βGal in cases 4, 8, 9, 10, 11, and 12. However, the peritoneum showed little increase in fluorescence over time in all cases. Thus, the fluorescence increase from the 0-min time point may be a more effective marker for diagnosis than the fluorescence intensity itself.

A major limitation of this study is that we used the comparison of primary tumor vs. peritoneum as a proxy for assessing peritoneal metastasis. As we have previously reported^19^, similar enzymatic activity cannot be guaranteed in metastases and primary tumors. In this study, the imaging of peritoneal metastatic nodules was carried out in only one case (Case 7). However, the peritoneum in all other cases showed little increase in fluorescence; case 7 showed a greater increase in fluorescence. Thus, we speculate that β-Gal is a promising target enzyme for detecting peritoneal metastasis in GC. To gather a sufficiently large number of cases with peritoneal metastasis from a single institution would take a long time; however, we plan to continue imaging peritoneal metastasis with SPiDER-βGal in the future to strengthen our observations.

Previously, 5-ALA was reported to be useful for the fluorescence imaging of peritoneal metastasis of GC^10,11^. In addition, ICG was shown to detect peritoneal metastasis of colorectal cancer^12^. Activatable fluorescence probes are novel tools in the field of fluorescence imaging, with advantages of convenient and non-invasive use, requiring simple topical spraying without preoperative preparation to obtain conclusive results. For the intraoperative investigation of the peritoneal cavity by laparoscopy or laparotomy, activatable fluorescence probes may be useful options for improving the diagnostic results of peritoneal metastasis.

Thus, we conclude that β-Gal is a promising target enzyme of an activatable fluorescence probe for imaging peritoneal metastasis in GC. Further, SPiDER-βGal can be applied to clinical samples. We believe that the results of this study significantly contribute to improving the clinical diagnostic results in GC patients.

## Methods

### Cell lines and culture

The following six human gastric cancer cell lines were used in this study: MKN28, MKN45, MKN74, Kato3, HGC27, and NUGC4. All cell lines were purchased from RIKEN cell bank (Ibaraki, Japan). HGC27 cells were cultured in minimum essential medium (Sigma, St. Louis, MO, USA), and the other cell lines were cultured in RPMI1640 (Gibco, MA, USA). Both culture media contained 10% fetal bovine serum (Gibco, Waltham, MA, USA), penicillin (100 U/mL), and streptomycin (100 μg/mL); all cells were cultured at 37 °C under 5% CO_2_.

### Preserved clinical samples

A total of 89 primary GC and 20 normal peritoneal (NP) samples were analyzed in this study. The GC samples were obtained from a total of 98 consecutive patients with GC who underwent surgery between January 2014 and December 2017 and whose samples could be collected. Nine patients were excluded because either the sample volume was too low to be used in experiments (n = 7) or they were diagnosed with neuroendocrine tumors (n = 1) or lymphoma (n = 1). We collected NP samples from 20 patients who underwent surgery for various benign or malignant diseases between May and June of 2018. All patients underwent surgery at the University Hospital of Kyoto Prefectural University of Medicine (KPUM), and written informed consent was obtained from each participant. Human samples were collected and preserved at − 80 °C. All investigations conformed with the tenets of the Declaration of Helsinki and the study was approved by the Institutional Review Board (IRB) of KPUM (ERB-C-67 for sample collection and ERB-C-1288 for assessing enzymatic activity).

### Preparation of cell lysates

To evaluate the enzymatic activity of β-Gal, we prepared cell and tissue lysates. For the preparation of the cell lysates, cultured cells were washed with phosphate-buffered saline (PBS), CelLytic M (Sigma) was added, and the cells were homogenized using an ultrasonic homogenizer (UH-50, SMT, Tokyo, Japan). Similarly, for the preparation of tissue lysates, CelLytic M was added, and the tissues were cut with scissors and then homogenized using an ultrasonic homogenizer. Both cell and tissue homogenates were centrifuged for 10 min at 14,000 × *g* at 4 °C. The supernatant was collected, and the protein concentration was quantified using the BCA method (Thermo Scientific, Waltham, MA, USA).

### Enzyme activity assay

For measuring GGT and DPPIV activity, we used gGlu-HMRG and EP-HMRG, which are GGT and DPPIV-activatable fluorescence probes, respectively. The GGT and DPPIV activity was measured in 384-well black plates with 5 μL/well lysate (1 mg/mL protein concentration) and 15 μL/well fluorescence probe solution (1.33 μM in pH 7.4 PBS, final concentration 1.0 μM). HMRG was used as the positive-control fluorophore. An EnVision multilabel plate reader (FITC filter; excitation/emission = 485 nm/535 nm) (Perkin Elmer, Waltham, MA, USA) was used to measure the fluorescence intensity for GGT activity. A GloMax Discover System (excitation/emission = 475 nm/ 500–550 nm) (Promega, Madison, WI, USA) was used for measuring DPPIV activity. The activity was calculated as follows: Activity = (Fluorescence increase rate)/(Fluorescence intensity of HMRG in lysate—Fluorescence intensity of gGlu-HMRG or EP-HMRG just after the addition of lysate)/(protein concentration).

For measuring β-Gal activity, we used the FluoReporter lacZ/Galactosidase Quantitation Kit (Molecular Probes, USA). The β-Gal activity was measured in 96-well black plates using 10 μL/well lysate (1 mg/mL protein concentration) and 100 μL/well 3-carboxy-umbelliferyl β-d-galactopyranoside solution (1.1 mM in pH 5.0 acetate buffer or pH 7.4 PBS, final concentration 1.0 mM). The plate was incubated at 37 °C for 30 min. After incubation, a stop buffer (0.2 M Na_2_CO_3,_ 50 μL/well) was added when the measurement was performed in a pH 5.0 acetate buffer; however, no stop buffer was added when the measurement was performed in pH 7.4 PBS. The fluorescence intensity (excitation/emission = 390 nm/460 nm) was measured using a microplate reader (SpectraMax M2e, Molecular Devices, Sunnyvale, CA, USA). The β-Gal activity was calculated as the ratio of the fluorescence intensity of each sample to that of the positive control (normalized fluorescence intensity); 0.1 mM 7-hydroxycoumarin-3-carboxylic acid was used as the positive-control fluorophore.

### Live-cell fluorescence imaging

Cells from each cell line were plated on a cover glass-bottomed culture well at a density of 1 × 10^5^ cells and incubated at 37 °C under 5% CO_2_ for 2 days. The cells were washed using Hanks’ balanced salt solution (HBSS, Wako) twice, and 1 μM SPiDER-βGal in HBSS was added to the cells and incubated for 15 min. Fluorescence images were captured using a fluorescence microscope (BZ-X810, KEYENCE, Japan). The TRITC filter set (excitation 545/25 nm, emission 605/70 nm, dichroic 565 nm) was used for fluorescence images; bright-field images were also acquired.

### Mouse model of peritoneal metastasis

All procedures with animals were carried out in compliance with the Manual for Animal Experiments and approved by the Kyoto Prefectural University of Medicine. BALB/c Ajcl-nu/nu 5-week-old female mice were purchased from SHIMIZU Laboratory Supplies Co., Ltd., Japan. A total of 1 × 10^6^ MKN45 and NUGC4 cells, suspended in PBS, were injected into the peritoneal cavity. Fluorescence imaging was performed 2–4 weeks after the injection of tumor cells. The study was carried out in compliance with the ARRIVE guidelines.

### Ex vivo fluorescence imaging study of GC model mice

To evaluate the fluorescence intensity of SPiDER-βGal, we performed fluorescence imaging of the mouse model as described above. Mice were sacrificed using deep anesthesia, and the mesentery and bowel were excised. SPiDER-βGal (50 μM in PBS, containing dimethyl sulfoxide as a co-solvent) was sprayed on the samples. Fluorescence images were obtained using an in vivo imaging system (IVIS Lumina Series III). Filter settings of excitation 520 nm and emission 570 nm were used for the detection of SPiDER after the enzymatic reaction. Fluorescence images were captured every 2 min using a sequence setup.

### Ex vivo imaging of fresh human samples

Thirteen GC patients who underwent various surgeries, including distal gastrectomy, proximal gastrectomy, total gastrectomy, and metastatic nodules resection, consecutively at the university hospital of KPUM and who provided written informed consent for this study were included for imaging analysis. Twelve primary tumor samples and one peritoneal metastatic sample were obtained. The resected samples were carried to the IVIS immediately, and fluorescence imaging was performed with SPiDER-βGal. We substituted the primary tumors vs. visceral peritoneum for imaging peritoneal metastasis because it would take a long time limited to the peritoneal metastasis samples. Both mucosa and peritoneum sides were imaged. Fluorescence intensity of the tumor and peritoneum was evaluated in the region of interest (ROI) with Living Image version 4.4 (PerkinElmer, MA, USA). This study was approved by the IRB of KPUM (approval number ERB-C-1470-1).

### Statistical analysis

Statistical analysis was performed using ystat 2008 software. The two-tailed Mann–Whitney *U* test was used to compare the β-Gal activity and fluorescence intensity in vitro and ex vivo. JMP 13.0 software (SAS Institute, Cary, NC, USA) was used for comparing the β-Gal activity for each clinicopathological factor with the Wilcoxon test and for drawing the receiver operating characteristic (ROC) curves. Results with *p* < 0.05 were considered to be statistically significant.

## Supplementary Information

Supplementary Information 1.

## Abbreviations

5-ALA: 5-Aminolevulinic acid
β-Gal: β-Galactosidase
AUC: Area under the ROC curve
BMI: Body mass index
CA19-9: Carbohydrate antigen 19-9
CEA: Carcinoembryonic antigen
CRP: C-reactive protein
CT: Computed tomography
DPPIV: Dipeptidyl peptidase IV
GC: Gastric cancer
GGT: Gamma-glutamyl transpeptidase
HBSS: Hanks’ balanced salt solution
HMRG: Hydroxymethyl rhodamine green
ICG: Indocyanine green
IRB: Institutional Review Board
KPUM: Kyoto Prefectural University of Medicine
NAC: Neoadjuvant chemotherapy
NP: Normal peritoneal
PBS: Phosphate-buffered saline
ROC: Receiver operating characteristic
ROI: Region of interest

## References

[1] Jemal A, Bray F, Center MM, Ferlay J, Ward E, Forman D (2011). Global cancer statistics. CA Cancer J. Clin..

[2] Japanese Gastric Cancer Association (2017). Japanese gastric cancer treatment guidelines 2014 (ver. 4). Gastric Cancer.

[3] Fujitani K, Yang HK, Mizusawa J, Kim YW, Terashima M, Han SU (2016). Gastrectomy plus chemotherapy versus chemotherapy alone for advanced gastric cancer with a single non-curable factor (REGATTA): a phase 3, randomised controlled trial. Lancet Oncol..

[4] Wang Z, Chen JQ (2011). Imaging in assessing hepatic and peritoneal metastases of gastric cancer: a systematic review. BMC Gastroenterol..

[5] Leake PA, Cardoso R, Seevaratnam R, Lourenco L, Helyer L, Mahar A (2012). A systematic review of the accuracy and indications for diagnostic laparoscopy prior to curative-intent resection of gastric cancer. Gastric Cancer.

[6] Hori Y, SAGES Guidelines Committee (2008). Diagnostic laparoscopy guidelines: this guideline was prepared by the SAGES Guidelines Committee and reviewed and approved by the Board of Governors of the Society of American Gastrointestinal and Endoscopic Surgeons (SAGES), November 2007. Surg. Endosc..

[7] Miki Y, Tokunaga M, Tanizawa Y, Bando E, Kawamura T, Terashima M (2015). Staging laparoscopy for patients with cM0, type 4, and large type 3 gastric cancer. World J. Surg..

[8] Hato S, Iwasaki Y, Mizusawa J, Terashima M, Katai H, Yoshikawa T (2017). Effectiveness and limitations of staging laparoscopy for peritoneal metastases in advanced gastric cancer from the results of JCOG0501: a randomized trial of gastrectomy with or without neoadjuvant chemotherapy for type 4 or large type 3 gastric cancer. Int. J. Clin. Oncol..

[9] Irino T, Sano T, Hiki N, Ohashi M, Nunobe S, Kumagai K (2018). Diagnostic staging laparoscopy in gastric cancer: a prospective cohort at a cancer institute in Japan. Surg. Endosc..

[10] Murayama Y, Ichikawa D, Koizumi N, Komatsu S, Shiozaki A, Kuriu Y (2012). Staging fluorescence laparoscopy for gastric cancer by using 5-aminolevulinic acid. Anticancer Res..

[11] Kishi K, Fujiwara Y, Yano M, Motoori M, Sugimura K, Ohue M (2014). Diagnostic laparoscopy with 5-aminolevulinic-acid-mediated photodynamic diagnosis enhances the detection of peritoneal micrometastases in advanced gastric cancer. Oncology.

[12] Liberale G, Vankerckhove S, Caldon MG, Ahmed B, Moreau M, Nakadi IE (2016). Fluorescence imaging after indocyanine green injection for detection of peritoneal metastases in patients undergoing cytoreductive surgery for peritoneal carcinomatosis from colorectal cancer: a pilot study. Ann. Surg..

[13] Urano Y, Sakabe M, Kosaka N, Ogawa M, Mitsunaga M, Asanuma D (2011). Rapid cancer detection by topically spraying a γ-glutamyltranspeptidase-activated fluorescent probe. Sci. Transl. Med..

[14] Onoyama H, Kamiya M, Kuriki Y, Komatsu T, Abe H, Tsuji Y (2016). Rapid and sensitive detection of early esophageal squamous cell carcinoma with fluorescence probe targeting dipeptidylpeptidase IV. Sci. Rep..

[15] Asanuma D, Sakabe M, Kamiya M, Yamamoto K, Hiratake J, Ogawa M (2015). Sensitive β-galactosidase-targeting fluorescence probe for visualizing small peritoneal metastatic tumours in vivo. Nat. Commun..

[16] Ueo H, Shinden Y, Tobo T, Gamachi A, Udo M, Komatsu H (2015). Rapid intraoperative visualization of breast lesions with γ-glutamyl hydroxymethyl rhodamine green. Sci. Rep..

[17] Hino H, Kamiya M, Kitano K, Mizuno K, Tanaka S, Nishiyama N (2016). Rapid cancer fluorescence imaging using a γ-glutamyltranspeptidase-specific probe for primary lung cancer. Transl. Oncol..

[18] Miyata Y, Ishizawa T, Kamiya M, Yamashita S, Hasegawa K, Ushiku A (2017). Intraoperative imaging of hepatic cancers using γ-glutamyltranspeptidase-specific fluorophore enabling real-time identification and estimation of recurrence. Sci. Rep..

[19] Kubo H, Hanaoka K, Kuriki Y, Komatsu T, Ueno T, Kojima R (2018). Rapid detection of metastatic lymph nodes of colorectal cancer with a gamma-glutamyl transpeptidase-activatable fluorescence probe. Sci. Rep..

[20] Doura T, Kamiya M, Obata F, Yamaguchi Y, Hiyama TY, Matsuda T (2016). Detection of LacZ-positive cells in living tissue with single-cell resolution. Angew. Chem. Int. Ed. Engl..

[21] Nakamura Y, Mochida A, Nagaya T, Okuyama S, Ogata F, Choyke PL (2017). A topically-sprayable, activatable fluorescent and retaining probe, SPiDER-βGal for detecting cancer: advantages of anchoring to cellular proteins after activation. Oncotarget.

[22] Bosmann HB, Hall TC (1974). Enzyme activity in invasive tumors of human breast and colon. Proc. Natl. Acad. Sci. U. S. A..

[23] Chatterjee SK, Bhattacharya M, Barlow JJ (1979). Glycosyltransferase and glycosidase activities in ovarian cancer patients. Cancer Res..

[24] Wielgat P, Walczuk U, Szajda S, Bień M, Zimnoch L, Mariak Z (2006). Activity of lysosomal exoglycosidases in human gliomas. J. Neurooncol..

[25] Dimri GP, Lee X, Basile G, Acosta M, Scott G, Roskelley C (1995). A biomarker that identifies senescent human cells in culture and in aging skin in vivo. Proc. Natl. Acad. Sci. U. S. A..

[26] Sugizaki T, Zhu S, Guo G, Matsumoto A, Zhao J, Endo M (2017). Treatment of diabetic mice with the SGLT2 inhibitor TA-1887 antagonizes diabetic cachexia and decreases mortality. NPJ. Aging Mech. Dis..

[27] Perez-Mancera PA, Young AR, Narita M (2014). Inside and out: the activities of senescence in cancer. Nat. Rev. Cancer.

