# Supplementary material for: β-Galactosidase is a target enzyme for detecting peritoneal metastasis of gastric cancer
Source: Sci Rep. 2021 May 21;11:10664. doi: 10.1038/s41598-021-88982-2 (PMC8139979; doi:10.1038/s41598-021-88982-2)
Supplement: Supplementary file 1 — Supplementary Information 1. [file 41598_2021_88982_MOESM1_ESM.docx]

**Supplementary information**

**β-Galactosidase is a target enzyme for detecting peritoneal metastasis of gastric cancer**

Hidemasa Kubo^1^, Yasutoshi Murayama^1,*^, Soichiro Ogawa^1^, Tatsuya Matsumoto^1^, Masayuki Yubakami^1^, Takuma Ohashi^1^, Takeshi Kubota^1^, Kazuma Okamoto^1^, Mako Kamiya^2^, Yasuteru Urano^2,3,4^, Eigo Otsuji^1^

^1^Division of Digestive Surgery, Department of Surgery, Kyoto Prefectural University of Medicine, 465 Kajii-cho, Kamigyo-ku, Kyoto 602-8566, Japan

^2^Graduate School of Medicine, The University of Tokyo, 7-3-1 Hongo, Bunkyo-ku, Tokyo 113-0033, Japan

^3^Graduate School of Pharmaceutical Sciences, The University of Tokyo, 7-3-1 Hongo, Bunkyo-ku, Tokyo 113-0033, Japan

^4^CREST (Japan) Agency for Medical Research and Development (AMED), 1-7-1 Otemachi, Chiyoda-ku, Tokyo 100-0004, Japan

***Corresponding Author:**

Yasutoshi Murayama

Postal address: Division of Digestive Surgery, Department of Surgery, Kyoto Prefectural University of Medicine, 465 Kajii-cho, Kamigyo-ku, Kyoto 602-8566, Japan

Phone: +81-75-251-5527

Fax.: +81-75-251-5522

E-mail: murayama@koto.kpu-m.ac.jp

**Figure and legends**

**Supplementary Figure 1**


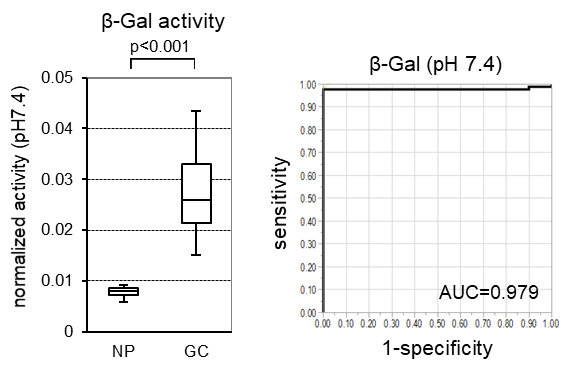


β-Gal activity of preserved GC (n=89) and NP (n=20) samples at pH 7.4. β-Gal; β-galactosidase, GC; gastric cancer, NP; normal peritoneum, AUC; area under the curve

**Supplementary Figure 2**


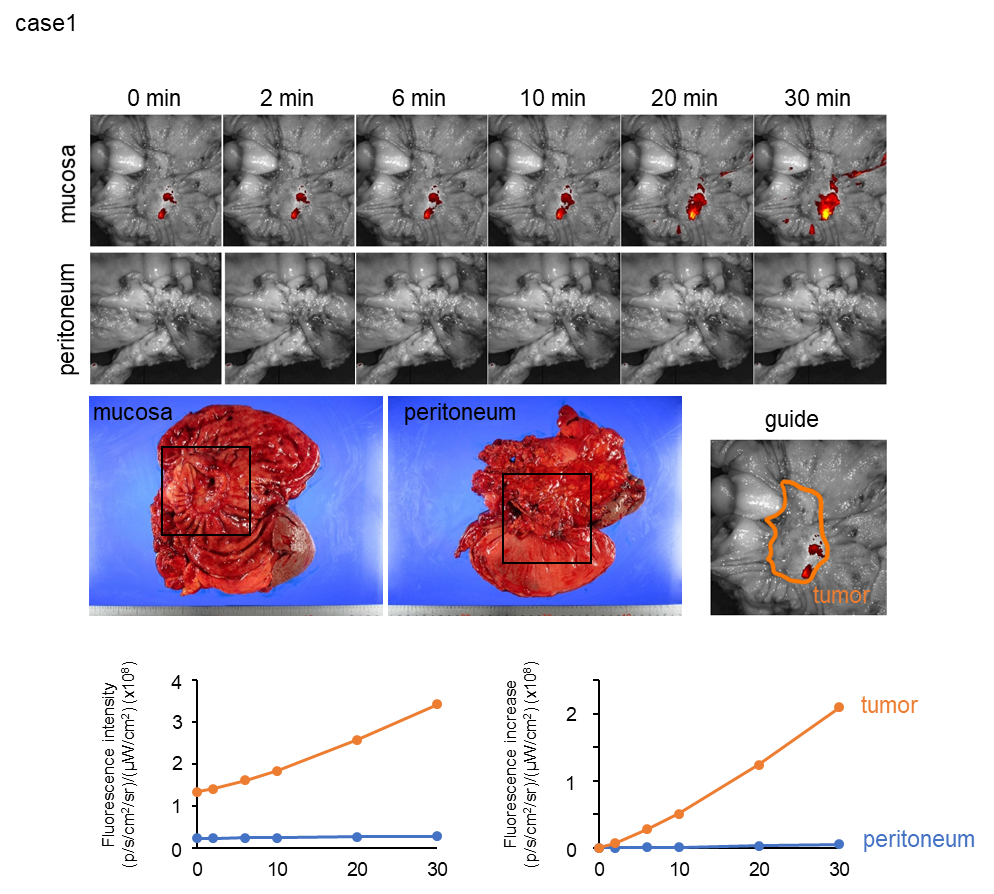


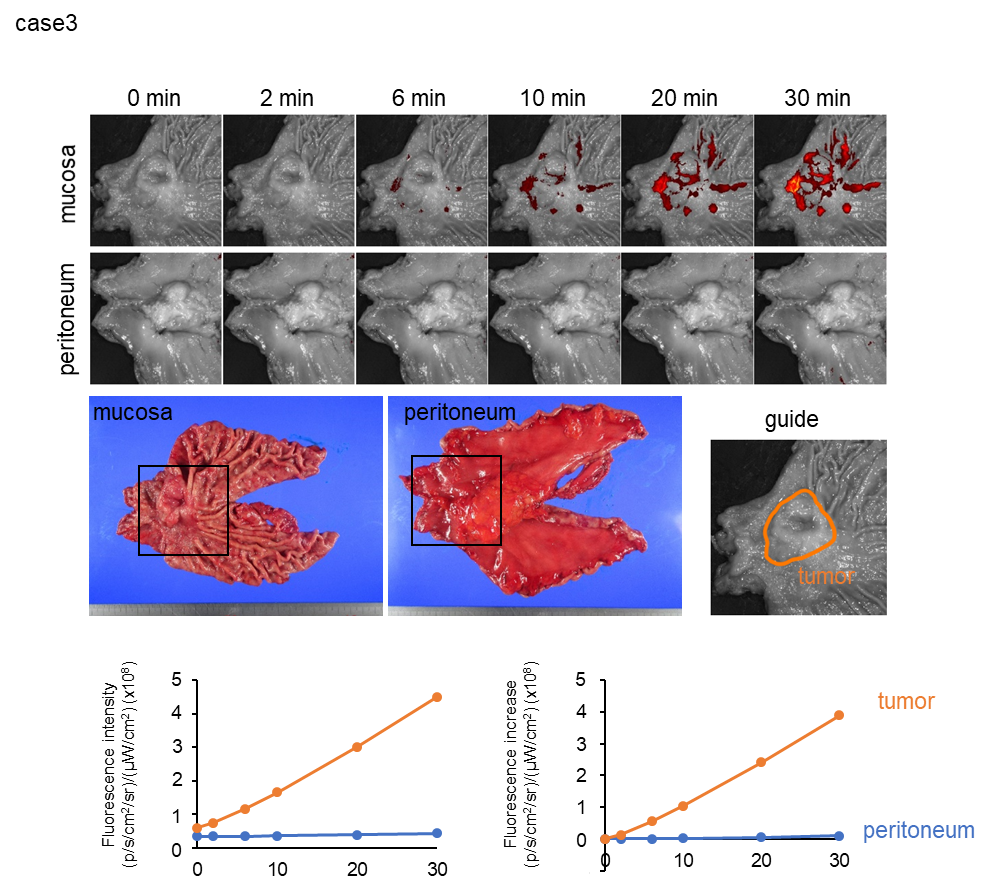


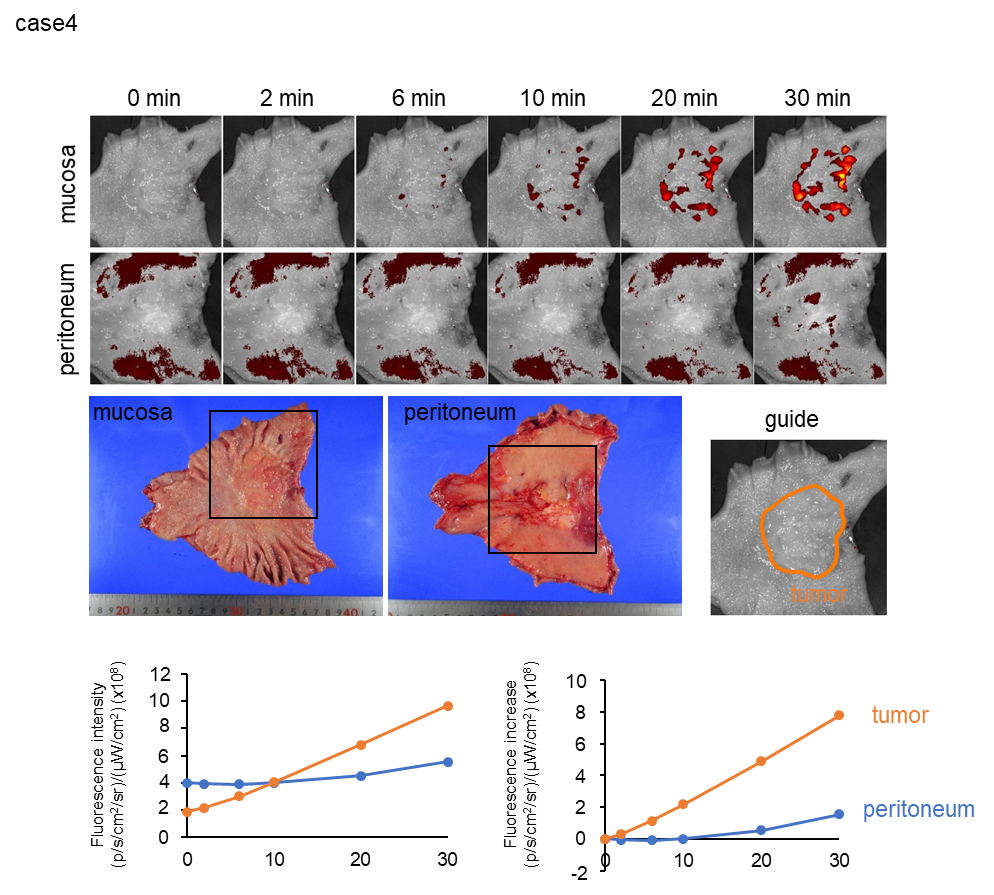


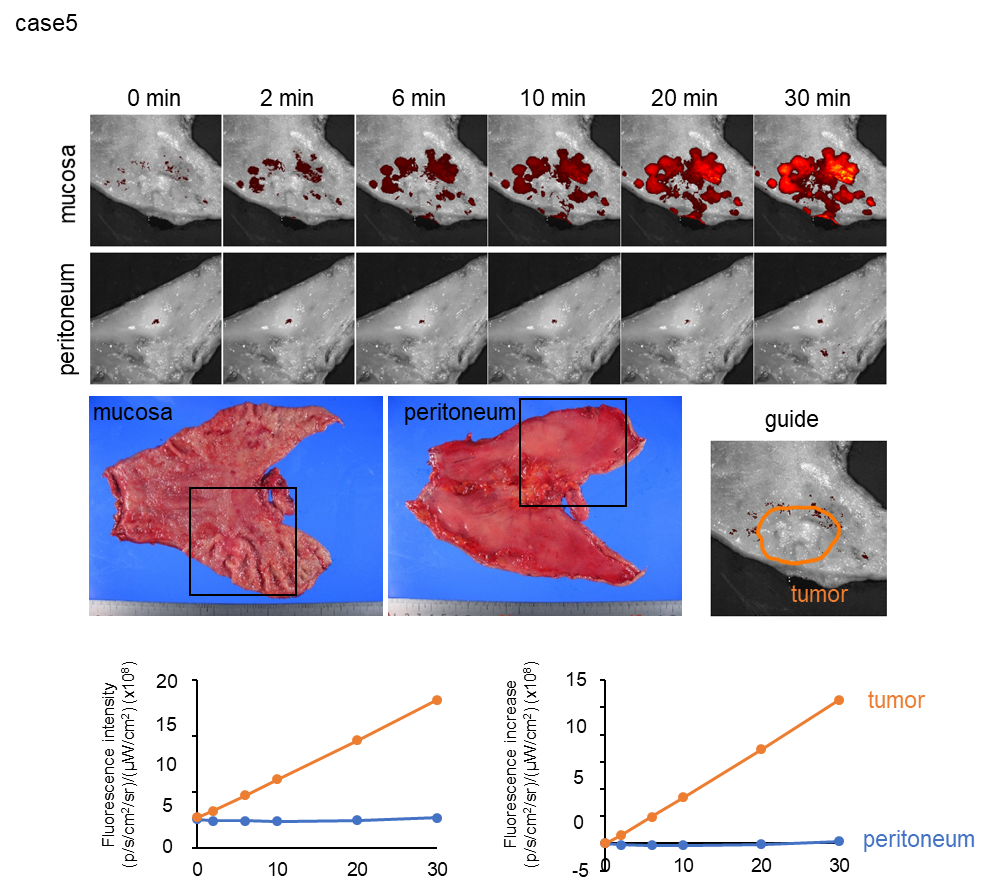


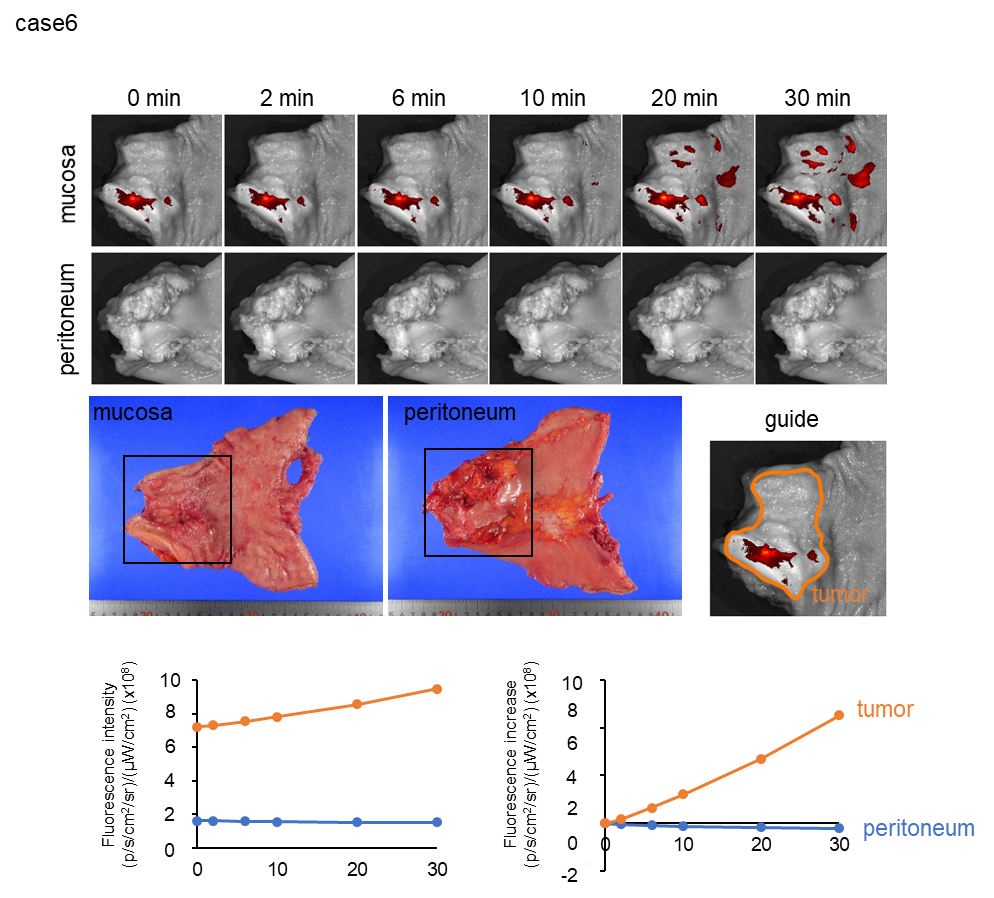


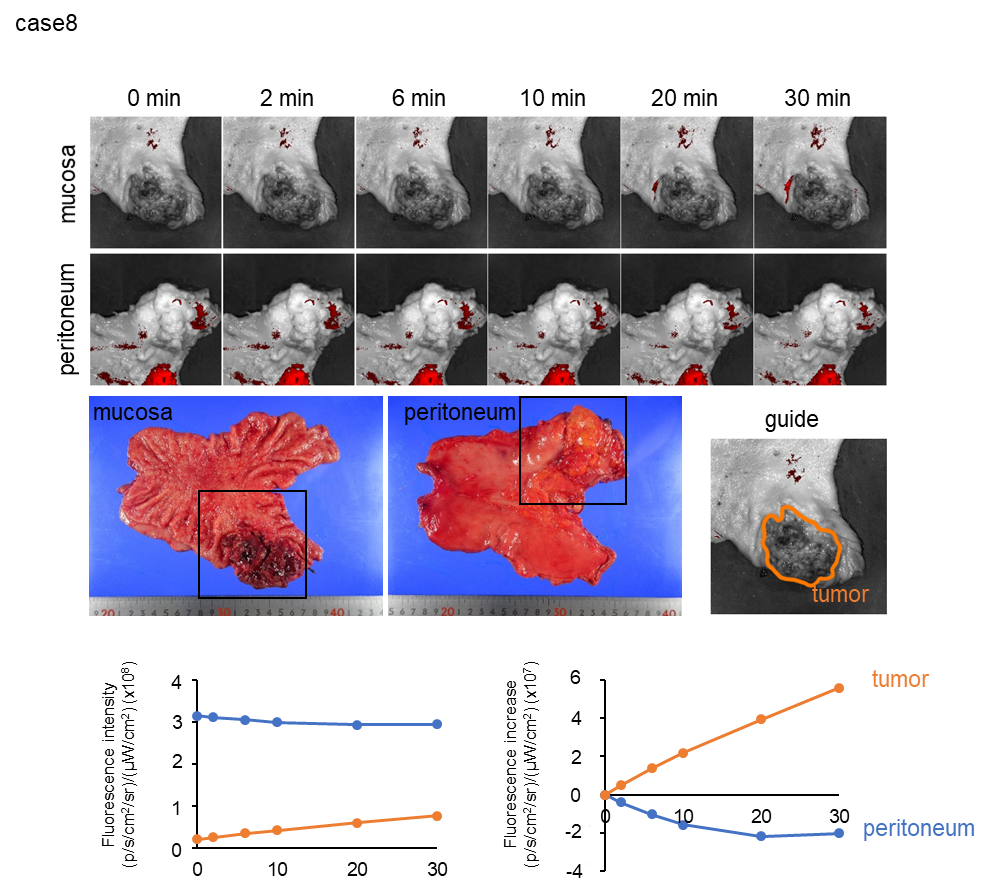


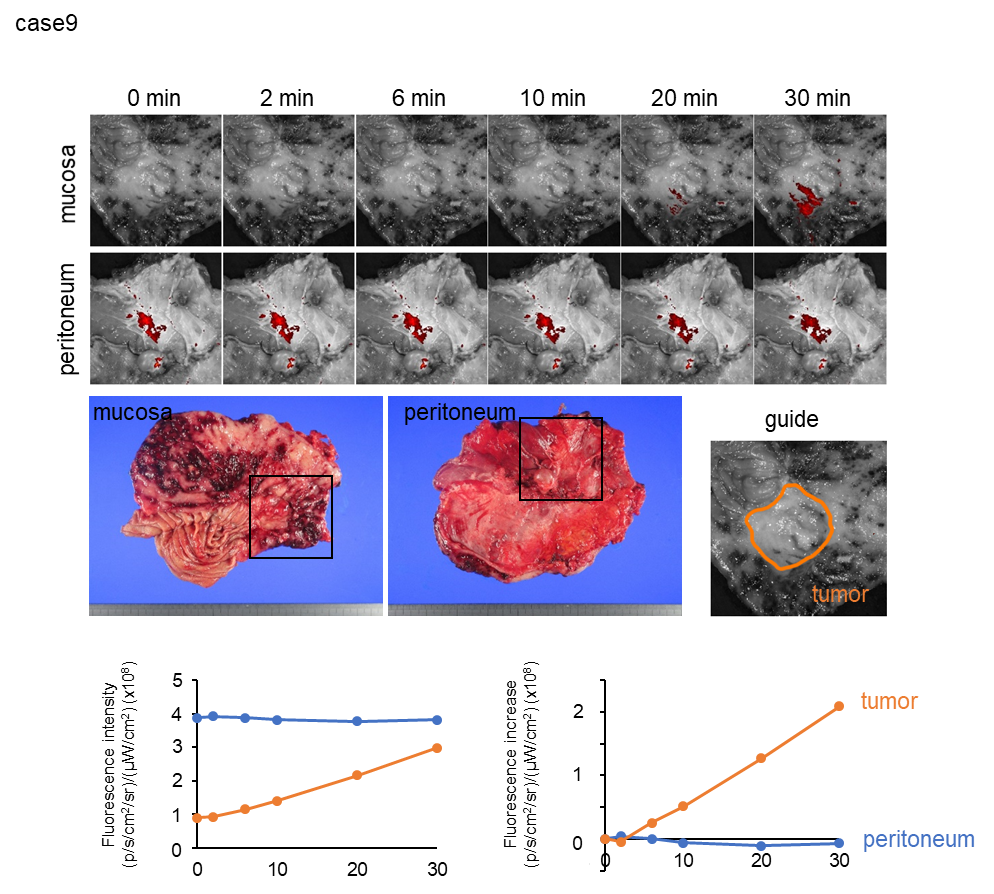


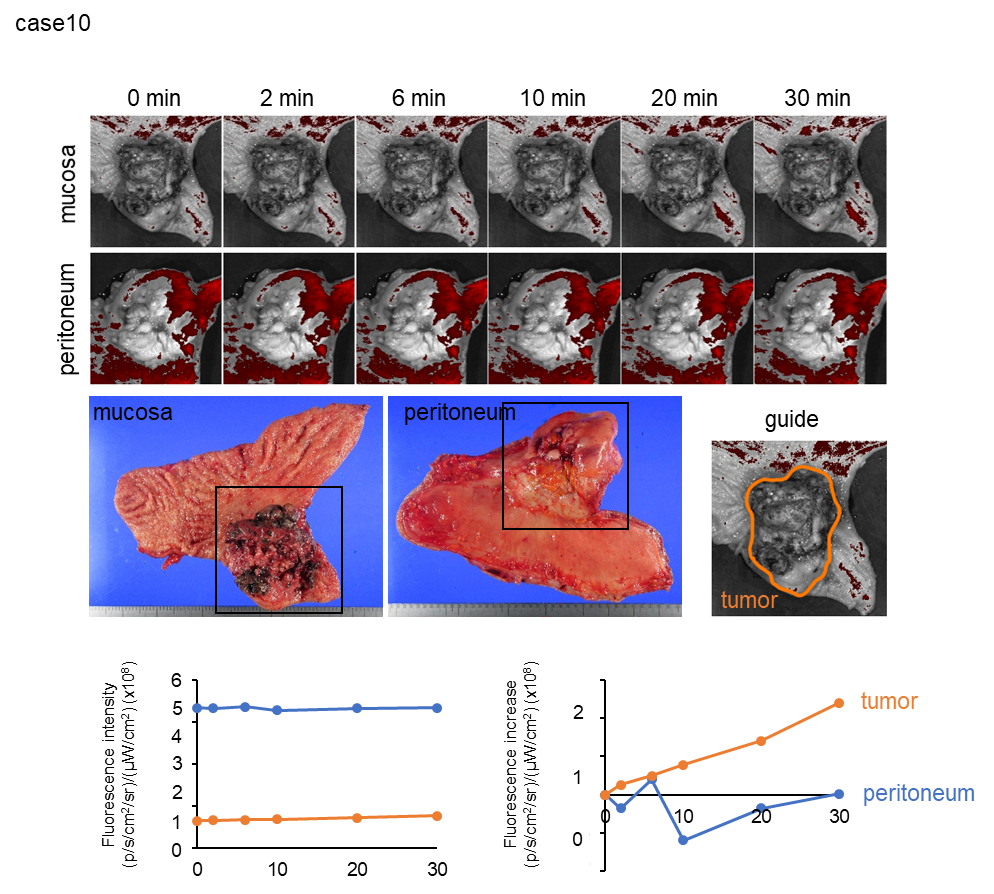


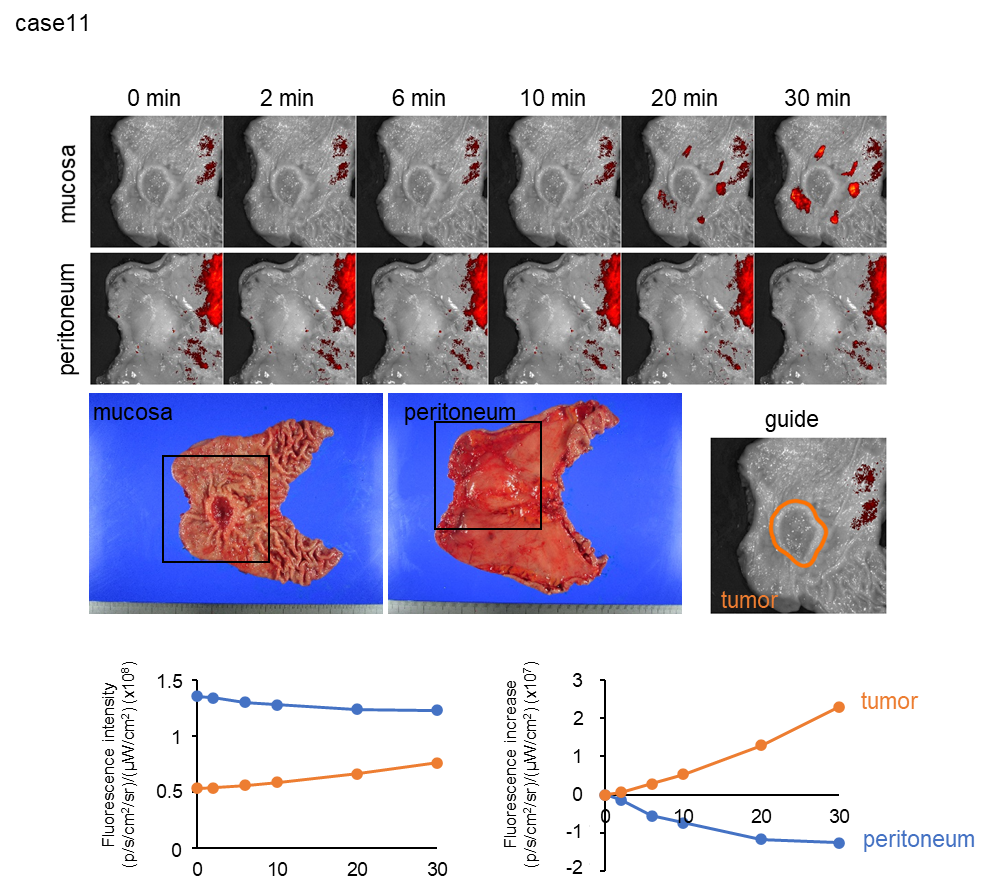


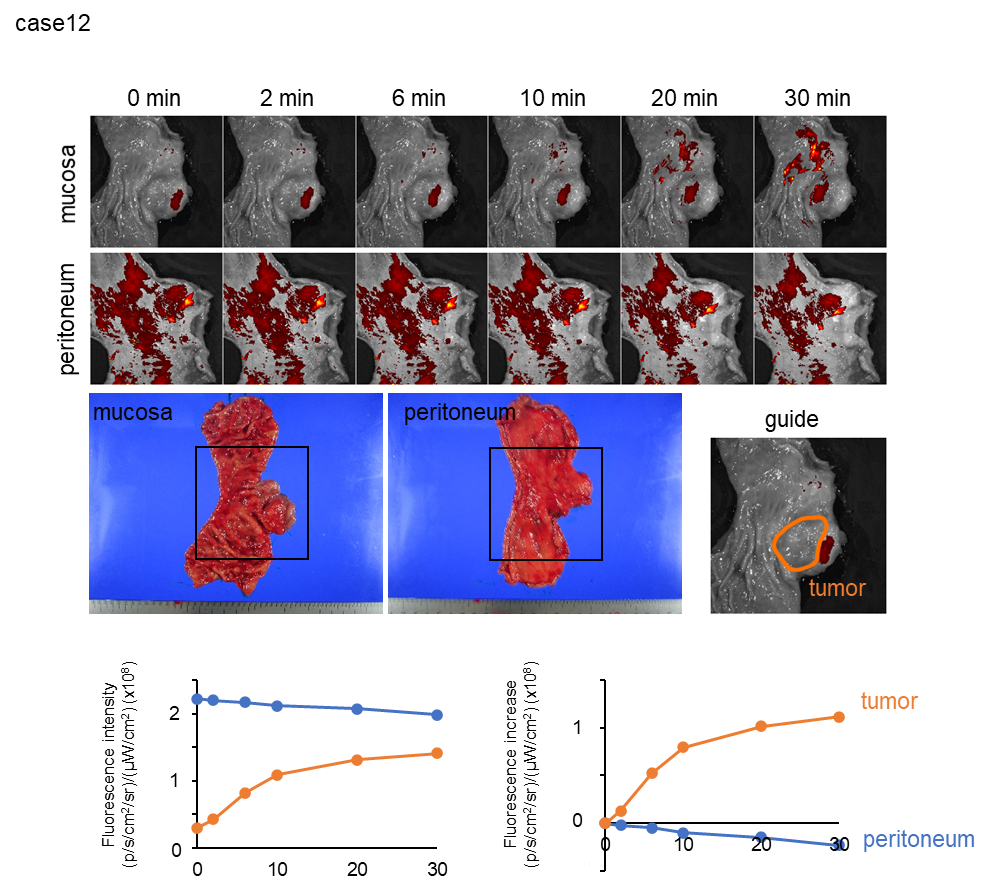


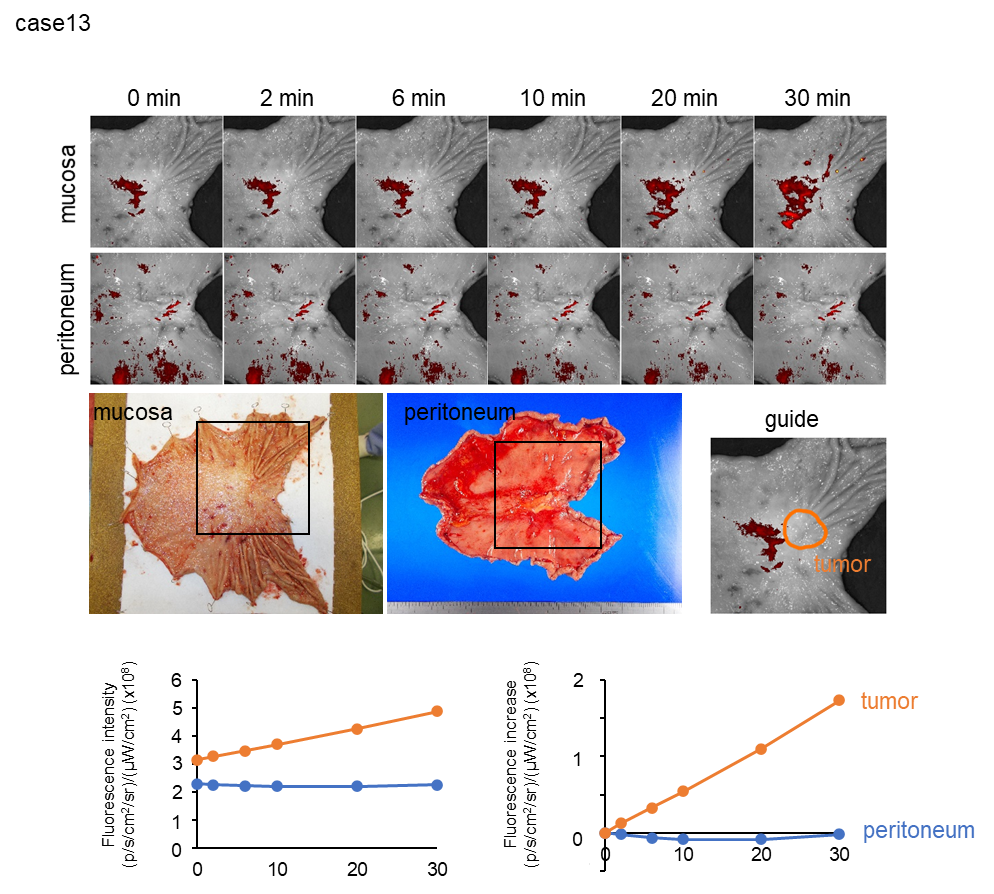


Fluorescence imaging of fresh human gastric cancer samples.
